# Supplementary material for: The causal relationship between the human gut microbiota and pyogenic arthritis: a Mendelian randomization study
Source: Front Cell Infect Microbiol. 2024 Nov 26;14:1452480. doi: 10.3389/fcimb.2024.1452480 (PMC11629706; doi:10.3389/fcimb.2024.1452480)
Supplement: Supplementary file 1 [file DataSheet1.zip › mendelian test/ebi-a-GCST90027622.csv_forest.pdf]

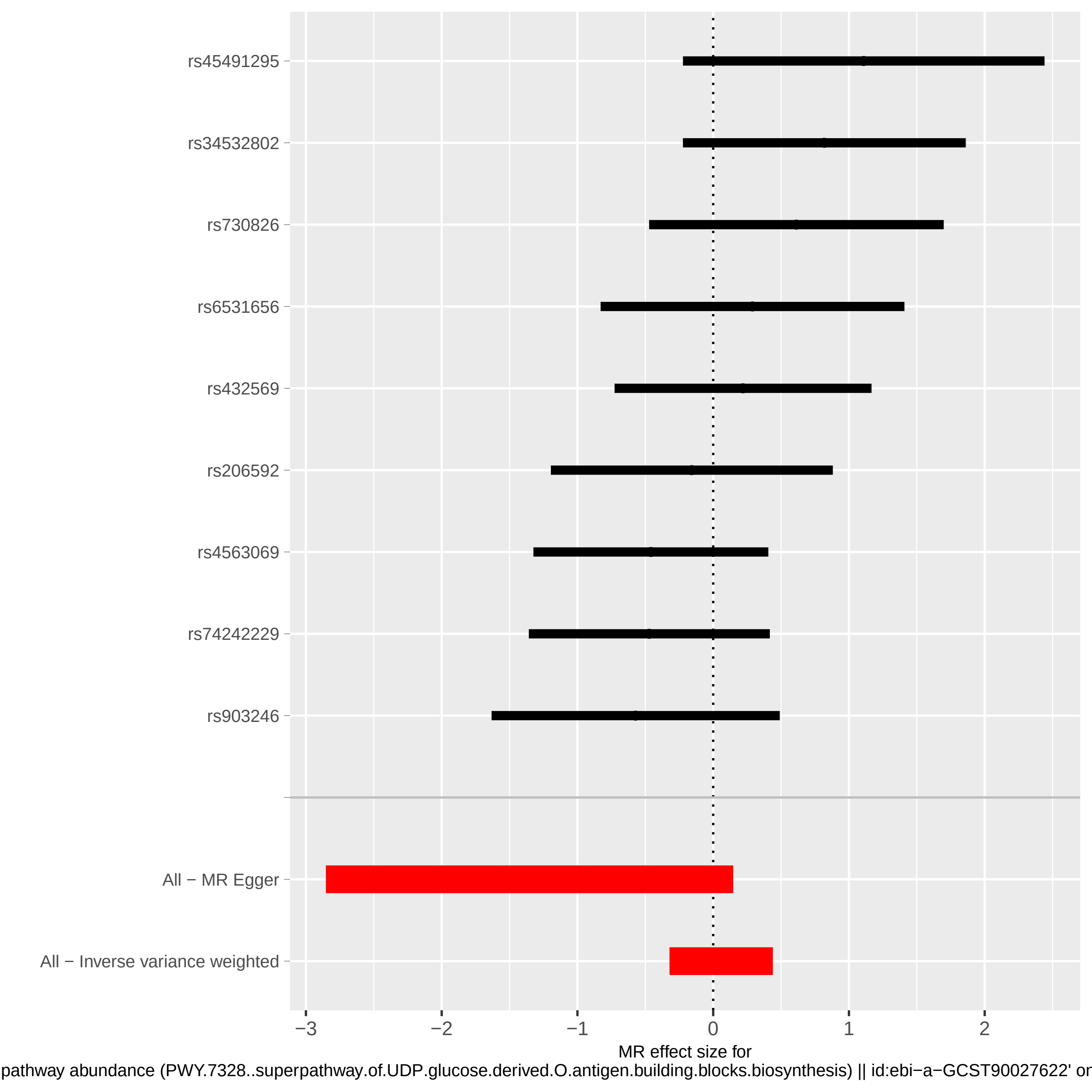
rs45491295
rs34532802
rs730826
rs6531656
rs432569
rs206592
rs4563069
rs74242229
rs903246
All − MR Egger
All − Inverse variance weighted
−3
−2
−1
0
1
2
MR effect size for
pathway abundance (PWY.7328..superpathway.of.UDP.glucose.derived.O.antigen.building.blocks.biosynthesis) || id:ebi−a−GCST90027622' on
